# High dose insulin therapy, an evidence based approach to beta blocker/calcium channel blocker toxicity

**DOI:** 10.1186/2008-2231-22-36

**Published:** 2014-04-08

**Authors:** Christina Woodward, Ali Pourmand, Maryann Mazer-Amirshahi

**Affiliations:** 1 Department of Emergency Medicine, George Washington University, Washington, DC 20037, USA

**Keywords:** High dose insulin, Beta-blocker, Calcium channel blocker, Overdose

## Abstract

Poison-induced cardiogenic shock (PICS) as a result of beta-blocker (β-blocker) or calcium channel blocker (CCB) overdose is a common and potentially life-threatening condition. Conventional therapies, including fluid resuscitation, atropine, cardiac pacing, calcium, glucagon, and vasopressors often fail to improve hemodynamic status. High-dose insulin (HDI) is an emerging therapeutic modality for PICS. In this article, we discuss the existing literature and highlight the therapeutic success and potential of HDI. Based on the current literature, which is limited primarily to case series and animal models, the authors conclude that HDI can be effective in restoring hemodynamic stability, and recommend considering its use in patients with PICS that is not responsive to traditional therapies. Future studies should be undertaken to determine the optimal dose and duration of therapy for HDI in PICS.

## Introduction

Poison-induced cardiogenic shock (PICS) due to beta-blocker (β-blocker) or calcium channel blocker (CCB) overdose is a frequent and potentially lethal occurrence [1]. β-blockers are used widely for conditions such as hypertension, dysrhythmias, and coronary artery disease; in the United States there were 128 million prescriptions for β-blockers filled in 2009 according to IMS Health, making them the fifth most commonly prescribed medication class in the country [2]. CCBs are also frequently utilized for patients with cardiovascular disease and other conditions, with an estimated 98 million prescriptions filled in 2010 alone [3]. The most common cause of PICS is β-blocker toxicity, with 24,465 exposures to β-blockers reported by the American Association of Poison Control Centers’ National Poison Data System in 2012 [4]. While less frequent, CCB overdose has been associated with the highest mortality rates amongst cardiovascular agents in the United States [5].

## Importance

Conventional therapies, including fluid resuscitation, atropine, cardiac pacing, calcium, glucagon, and vasopressors often fail to improve hemodynamic status in PICS secondary to β-blocker and CCB overdose. Even if there is a response to such traditional therapies, the response is often transient in nature [6-8]. High-dose insulin (HDI) (using doses 10-fold greater than traditional therapy for hyperglycemia) is an emerging therapeutic modality for PICS. Although there is a paucity of clinical trials and existing data are currently limited to case series or animal models, HDI has shown great promise as an effective treatment for β-blocker and CCB toxicity [7,9-13].

Unlike other countries such as Iran, where there is a growing trend to treat acutely poisoned patients in specialized tertiary hospitals where the clinical staff is trained specifically in toxicology, many poisoned patients in the U.S. are not seen at the bedside by a medical toxicologist [14]. As such, it is of critical importance that emergency medicine physicians, intensivists, and support staff (nursing, pharmacy) be equipped with the knowledge and comfort to utilize HDI. HDI is a potentially life-saving therapeutic option; however, its popularity is not yet widespread and has been mainly restricted to use as a rescue therapy after conventional methods fail [15]; this limitation is likely due to a lack of randomized, controlled trials and practitioner unfamiliarity. In this paper, we will review the existing literature and highlight the therapeutic success and potential of HDI.

## Mechanism of toxicity

β-blockers act on beta-receptors through competitive inhibition, indirectly decreasing the production of cAMP and thereby limiting calcium influx through L-type calcium channels with a resulting negative effect on heart rate and cardiac contractility [1,16,17]. CCBs exert their therapeutic and toxic effects by the direct blockade of L-type calcium channels causing relaxation of the vascular smooth muscle with subsequent vasodilation, and in the case of verapamil and diltiazem, inhibition of the sinoatrial and atrioventricular nodes. Calcium channel blockade concurrently triggers the heart to switch to preferential carbohydrate metabolism as opposed to the free fatty acid oxidation that occurs in the myocardium in the non-stressed state [8,17-19]. The effects of calcium channel antagonism are also seen in other parts of the body [1,6,17]. For example, in the beta-islet cells of the pancreas, calcium channel antagonism inhibits insulin secretion, producing insulin resistance and hyperglycemia [8,17,18].

Calcium flux is crucial to many aspects of normal myocardial activity, including contractility, pacemaker function, and signal propagation. Both CCBs and β-blockers ultimately result in decreased myocardial cytosolic calcium [1,17]. Life- threatening cardiovascular effects such as profound vasodilation with decreased systemic vascular resistance, bradycardia, conduction delay, hypotension, and resulting cardiogenic shock have been well established in BB and CCB overdose [17,20,21]. Other adverse effects include hyperglycemia (more common in CCB overdose) and lactic acid accumulation leading to metabolic acidosis [6,9,22,23]. In addition, altered mental status, dysrhythmias, seizures and other adverse effects may occur depending upon the specific agent ingested [17].

## Management of PCIS

The primary goal in the management of PCIS is to restore hemodynamic stability [9]. Treatment tends to be physician dependent, given the lack of clinical trials and established guidelines. Once the patients’ airway, breathing, and circulation have been stabilized, initial therapy for CCB and β-blocker overdose generally includes aggressive gastrointestinal decontamination. Activated charcoal, gastric lavage, and whole-bowel irrigation are potential options for decontamination in hemodynamically stable patients; however, these therapies are relatively contraindicated in the setting of shock. In patients with evidence of cardiovascular compromise, intravenous crystalloids such as normal saline, are initially administered as a bolus. Patients with symptomatic bradycardia can be treated with atropine and cardiac pacing; however, patients with β-blocker and CCB overdose often do not respond to these interventions [17]. Calcium and glucagon administration are often attempted as part of initial resuscitation efforts. Catecholamines, such as norepinephrine, can be administered as hemodynamic status warrants. For patients who remain hemodynamically unstable after these initial therapies, second line treatment options include HDI, lipid emulsion therapy [24,25] and mechanical life support (including intra-aortic balloon pump, cardiopulmonary bypass, or extracorporeal membrane oxygenation) [1].

## Mechanism of action

HDI has been postulated to improve hemodynamics in CCB and β-blocker overdose by several different mechanisms. Most notably, a number of studies have demonstrated that insulin administered in higher doses has strong positive inotropic properties [9,11,17,18,20,21,26]. HDI also assists myocardial uptake of carbohydrates, which is the preferred fuel substrate of the heart under stressed conditions [1,8,21,27] HDI also inhibits free fatty acid metabolism [27,28]. Additionally, exogenous insulin administration can help to overcome the insulin resistance and insulin deficiency that occurs in CCB toxicity [1]. HDI produces vasodilation, which improves local microcirculation [11,21] and aids systemic perfusion [9,11,20]. Studies have demonstrated accelerated oxidation of myocardial lactate and reversal of metabolic acidosis with HDI [9,26]. Response to catecholamines is also improved with addition of HDI [9]. While conventional therapy sometimes offers temporary improvement in hemodynamics, the hemodynamic stability achieved with HDI does not appear to be as transient in nature [21].

## Dosing and administration

There are no official guidelines regarding insulin dosing in PICS and wide practice variation exists. However, one of the most common recommendations consists of a 1 unit/kg bolus dose followed by a continuous infusion at 0.5-1 unit/kg per hour, which can be titrated to response. Insulin doses up to 10 units/kg per hour have been successfully used to treat PCIS [29]. A dextrose bolus of 0.5 g/kg can be administered with the initial insulin bolus in patients whose blood glucose is less than 400 mg/dL. A continuous dextrose infusion (0.5 g/kg per hour) should be initiated. It is preferable to administered concentrated dextrose solutions through a central venous catheter [17]. Supplemental intravenous dextrose (110–150 mg/dL or 6–8 mmol/L) can be administered as needed to maintain euglycemia; however, patients with CCB overdose may be hyperglycemic despite HDI [9,15,21]. Blood glucose should be checked every twenty minutes during the first hour, and can then be checked hourly [9], with the goal of maintaining blood glucose levels in the upper range of normal [15]. The onset of action of HDI is thought to be 15–45 minutes, but may be delayed several hours [15,21]. Once initiated, HDI therapy is continued until hemodynamic stability is achieved. There are no established recommendations regarding the proper duration of therapy and treatment with HDI should be guided by the patient’s hemodynamic status [21], with the goal of maintaining a heart rate of at least 50 beats/min and a systolic blood pressure of at least 100 mm Hg [9]. Case reports have documented variable duration of HDI, ranging from 9 hours to 49 hours [7,10,30].

## Adverse effects

HDI is relatively well tolerated; the most common adverse effects include hypoglycemia and hypokalemia; however, these are rare and reversible when proper serum monitoring and replacement is undertaken [21,31]. Supplemental glucose is often required throughout the administration of HDI and for as long as 24 hours after cessation of therapy [21]. Some adult patients may need up to 30 g of supplemental glucose per hour to maintain normokalemia, in addition to potassium supplements [15]. Serum potassium should be checked hourly during insulin titration and may be extended to every 6 h following titration and electrolyte stability [21]. Intravenous potassium repletion is required when concentrations drop below 2.8 mEq/L [21], with a target goal of maintaining concentrations at 2.8-3.2 mEq/L [9]. In some case reports, patients have not required potassium supplementation at all [7], while other authors have described the need for potassium supplementation averaging 2.7 mmol/h (or 4.1 mmol per 100 units of insulin) [31]. Hypokalemia during HDI is representative of intracellular shifting of potassium as opposed to total body depletion [21]. Practitioners can observe hypokalemia through electrocardiogram changes, beginning with decreased T-wave amplitude and progressing to ST segment depression, T-wave inversion, prolongation of the PR interval, increased P wave amplitude, and U wave appearance [32]. Despite the known risk of adverse arrhythmias with hypokalemia, there have been few such events recorded in the literature when HDI is used in cases of PICS [21]. It is important to note that the adverse effects associated with HDI are also present with the use of insulin at regular doses, which physicians, pharmacists, and nurses are accustomed to addressing in conditions such as diabetic ketoacidosis.

## Experimental and clinical data

Although prospective clinical studies in human subjects comparing the efficacy of HDI to conventional treatments are lacking, several experimental animal studies have demonstrated the utility of HDI in achieving hemodynamic stability. A series of studies in canines by Kline et al. consistently demonstrated improved inotropy when HDI was given after verapamil overdose [8,18,20,26]. In other studies, HDI successfully normalized heart rate [13] and reversed negative inotropy caused by propranolol overdose in canines [11,13]. Superior survival rates have been witnessed in various animal studies when HDI is administered in PICS [8,12,20,26,33]. In humans, several case reports have documented successful hemodynamic stabilization with insulin after initial failed treatment attempts with conventional therapy [6,7,25,34,35].

To date, there are no studies on the most appropriate way to wean HDI once hemodynamic stability has been achieved [21]. While some physicians opt for a slow taper, others advocate for abrupt cessation of HDI infusion as this method has been postulated to allow insulin concentrations to self-taper as lipid stores slowly release insulin [7,21]. Several case reports have documented worsening hypotension in patients with CCB overdose with early insulin withdrawal that was alleviated when the insulin infusion was subsequently increased [7,30]. In one reported case of verapamil overdose resulting in hypotension and a junctional rhythm, HDI was initiated 3.5 hours after presentation (0.5 IU/kg bolus and infusion at 0.5 IU/kg/h) following failure of blood pressure stabilization with intravenous fluids and metaraminol boluses, with subsequent improvement in blood pressure and conversion to normal sinus rhythm within 30 minutes [30]. Following abrupt termination of HDI 5.5 hours after presentation the patient again became hypotensive, therefore HDI was restarted at 8.5 hours as well as an adrenaline infusion which again achieved hemodynamic stability. HDI was continued until 30.5 hours and the patient remained stable throughout this time. In another case report of verapamil overdose resulting in initial hypotension and third degree heart block, HDI up to 70 units per hour was initiated after 45 minutes of refractory hypotension despite calcium chloride and glucagon, with subsequent improvement in blood pressure [7]. Eight hours after presentation, HDI was gradually weaned while maintaining glucagon and dopamine infusions, resulting in recurrent hypotension, which was improved when the dose of HDI was increased. HDI was continued for a total of 27.5 hours.

Treatment difficulty could be due to delayed initiation of insulin therapy, as rare case reports have documented treatment failure with HDI when initiated late, for example at the end of cardiopulmonary resuscitation or following multiple hours of alternative therapies [36,37]. Although many sources recommend a 1 unit/kg bolus dose followed by a continuous infusion at 0.5-1 unit/kg per hour [9,15,21], no ceiling effect has ever been established and higher doses have been postulated to be more effective [38], with good outcomes documented in patients receiving insulin boluses as high as 10 U/kg [39] and infusions as high as 22 U/kg/h [25].

## Conclusions

Although clinical trial data in humans are lacking, available published reports suggest that HDI is effective at restoring hemodynamic stability in CCB and β-blocker overdose. As such, HDI should be considered in patients with PCIS who do not respond to traditional therapies and providers who care for poisoned patients should be familiar with this potentially life-saving therapy. Future studies should be undertaken to determine the optimal dosing regimen and duration of therapy for HDI in PICS.

## Competing interests

The authors have no commercial associations or sources of support that might pose a conflict of interest.

## Authors’ contribution

All authors have made substantive contributions to the study, and all authors endorse the data and conclusions. All authors read and approved the final manuscript.
